# Mental Efficiency

**Published:** 1916-01

**Authors:** 


### The Department of Eugenics, Mental and Social Hygiene

CONDUCTED BY
DR. MALONE DUGGAN
813-814 Gibbs Building, San Antonio, Texas
All communications for this department should be sent to DR. DUGGAN

### Mental Efficiency.

The fire burning in our grate sends forth but a small fraction of its energy. The rushing stream empties its turbulent waters, unused, into the endless sea. Torrential rains deplete the fields of their greatest fertility. Merciless conflagrations consume boundless forests. In fact, wherever we turn in the whole realm of nature, there is to be seen this profligate waste. Much more extravagant than all this is the wanton waste of mental efficiency,—an energy no less dynamic than the pent up forces of nature. The normal human mind, capable of enormous development, rarely exceeds one-twelfth of its endowed capacity. It would be difficult to estimate this great loss to the value of life.

In the daily whirl of business affairs, efficiency and economy are the keynotes to success, though the exaggeration of these forces may lead to the reverse of the object sought for. In the acquirement of this success, the nervous strain is too great and the balance of the nervous equilibrium is broken. It is no common experience to find men and women in every walk of life, in every vocation or business, complaining of over-fatigue. With tired brains, they perform their duty imperfectly, which is the cause of their inefficiency. The trouble lies not so much in the system or amount of work performed, but in the manner in which the mind is trained to meet these emergencies. Faulty habits of training lead to waste of energy, which in turn cause the overstrain. If we were traveling to a distant city and were to come to the forks of the road, it would behoove us to first inquire which is the right road before proceeding on our journey. So, in forming habits of thinking, if we took the same pains to know that the training was done correctly, we would not be carried off in inefficient action.

Consciousness may be likened to a stream of mental energy, varying in intensity. The impression made in consciousness depends upon the sensitiveness of the perception,—the intensity with which attention is given. If the attention is continuous or strained, the impression will be weak and the judgment correspondingly shallow. If, on the other hand, the attention is keen and alert, fixed at the proper time and in the proper manner, the impression will be strong and lasting. This exercise of the attention is followed by periods of rest, just as the muscle must first relax before it can again contract.

Most of our actions are performed automatically. By far the larger number of the functions of the body are carried on by reflex action. No progress can be made in any education until first the elementary steps have become subconscious; i. e., performed without conscious effort. As an illustration, take the musician or typist, the mechanical performance of the hands in manipulating the keys is done unconsciously, leaving the conscious mind to be engrossed with its new experience, feelings and thoughts. Thus useful systems of mental automatisms are essential to proper habit formation and in this way make possible the expansion of mental endowment. When established in progression, with the mental development, they assure the strongest and most influential intellects.

It is an old saying that you should "know a little of everything, and much of something," which is to emphasize the necessity of specializing in some one thing. A general knowledge of things and people around you is requisite to a good conversational faculty, but to be of real use one must possess a special knowledge of some one thing. Just as the faculty of attention requires corresponding periods of relaxation to make it efficient, so its continuous usefulness demands that this relaxation be such that the attention will never became fagged; i. e., our mental activities should be so ordered that long vacation periods of rest would not be required. Intense mental activity for short periods with corresponding periods of relaxation is preferable to long strain or overexertion with long periods of recuperation.

With well fixed habits of attention and rest established, we should undertake only that for which we are prepared; limit our requirements only to that which we actually need, and then fix, by constant use, that which we have acquired. The mind so trained will be best fitted to reap the larger measure of its endowment.

---

Waco has let the contract for the building of a new city hospital to replace the frame building which was burned several weeks ago.

